# Intranodal palisaded myofibroblastoma in the submandibular gland region: a case report

**DOI:** 10.3389/fonc.2024.1362090

**Published:** 2024-07-31

**Authors:** Han Zhang, Min Wang, Liang Li, Shuo Shao, Ning Zheng

**Affiliations:** ^1^ Clinical Medical College, Jining Medical University, Jining, Shandong, China; ^2^ Department of Radiology, Jining No. 1 People’s Hospital affiliated to Shandong First Medical University, Jining, Shandong, China; ^3^ Department of Pathology, Jining No. 1 People’s Hospital affiliated to Shandong First Medical University, Jining, Shandong, China

**Keywords:** intranodal palisaded myofibroblastoma, submandibular gland, lymph node, magnetic resonance imaging, pathology, case report

## Abstract

Intranodal palisaded myofibroblastoma (IPM) is a rare benign tumor of the lymph nodes, particularly in inguinal lymph nodes. IPM originating from the submandibular gland lymph nodes is rarely encountered in clinical practice. Herein, we report the case of a 31-year-old male patient with IPM of the submandibular gland region and describe in detail magnetic resonance imaging findings and pathology. Magnetic resonance imaging detected a heterogeneous lesion with a hypointense rim on T2-weighted imaging with specificity in the left submandibular gland region. This case report will contribute to the accumulation of experience in the diagnosis of this disease.

## 1 Introduction

Intranodal palisaded myofibroblastoma (IPM) in the lymph nodes, also known as intranodal hemorrhagic spindle-cell tumor with amianthoid fibers, is a rare benign primary mesenchymal tumor that originates from differentiated smooth muscle cells and myofibroblasts in the lymph nodes. These tumors are most commonly found in inguinal lymph nodes. Although they can also occur in other locations, such as mediastinal, axillary, and submandibular lymph nodes (1), they are rarely encountered and easily misdiagnosed. At present, our understanding of its imaging manifestations is limited. Usually, IPM can be diagnosed by ultrasound (US)-guided biopsy.

This report presents a unique case of submandibular gland lymph nodes with specific magnetic resonance imaging (MRI) findings, which contributes to the existing knowledge base. In this case, the patient was treated with surgery at Jining No.1 People’s Hospital, Shandong Province. The patient was followed up for 18 months after surgery, and no signs of recurrence were observed. According to the current literature, as a benign tumor, IPM in the lymph nodes can be cured by surgical resection, with an approximately 6% recurrence rate and no malignant transformation, and local recurrence is very rare (2–4). We predict that the prognosis of our patient should be no recurrence and canceration.

## 2 Case description

A 31-year-old male patient presented with a painless mass in the left submaxillary region and was referred to our hospital. The mass was approximately 2 cm× 2cm× 2 cm in size when it was first discovered two years ago, and the lesion continued to enlarge. On physical examination, the left mandibular angle area was slightly swollen, and a 5 cm× 4 cm× 3.5 cm mass could be touched. And it was firm with no tenderness and poorly mobile. The surface skin was intact, with no ulceration or bleeding. On oral cavity examination, no other abnormal findings were noticed.

**Figure 1:**
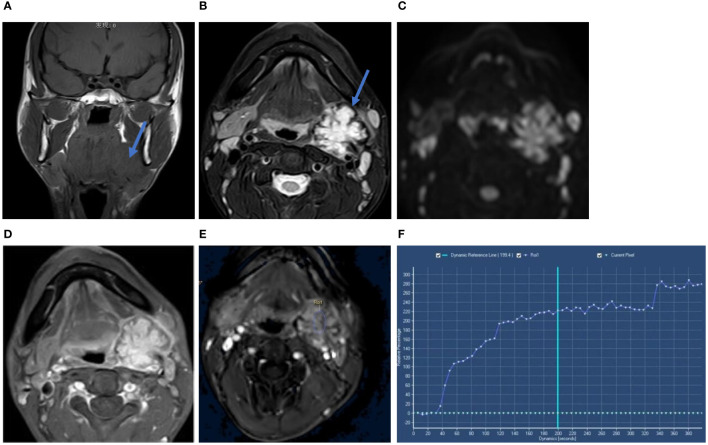
Coronal T1WI scan **(A)** showed the tumor mass on the upper pole of the left submandibular gland (blue arow). Axial T2WI-FS **(B)** showed heterogeneous hyperintense with a hypointense lobulated rim (blue arow). The tumor showed heterogeneous high signal intensity on DWI **(C)**. Axial gadolinium-enhanced T1-weighted image **(D)** showed heterogeneous enhancement with a hypointense rim. Time-intensity curve (TIC) was progressive ascending type **(E, F)**. Its x-axis represents the time of the enhanced scan, from 0 seconds to 380 seconds, with the extension of time, the curve is on the rise.

MRI showed a mass on the upper pole of the left submandibular gland, which appears isointense or hypointense on unenhanced T1-weighted imaging (T1WI) (**Figure 1A**) and heterogeneous hyperintense with a hypointense lobulated rim on T2-weighted imaging (T2WI) (**Figure 1B**). The tumor showed heterogeneous high signal intensity on diffusion-weighted imaging (DWI) (**Figure 1C**) and slightly low signal intensity on the apparent diffusion coefficient (ADC) map. The gadolinium-enhanced T1WI showed heterogeneous enhancement with an edge low signal enhancement band (**Figure 1D**), and the time–intensity curve (TIC) was of the progressive ascending type (**Figures 1E, F**). The adjacent submandibular glandular tissue and external carotid artery were compressed.

**Figure 2:**
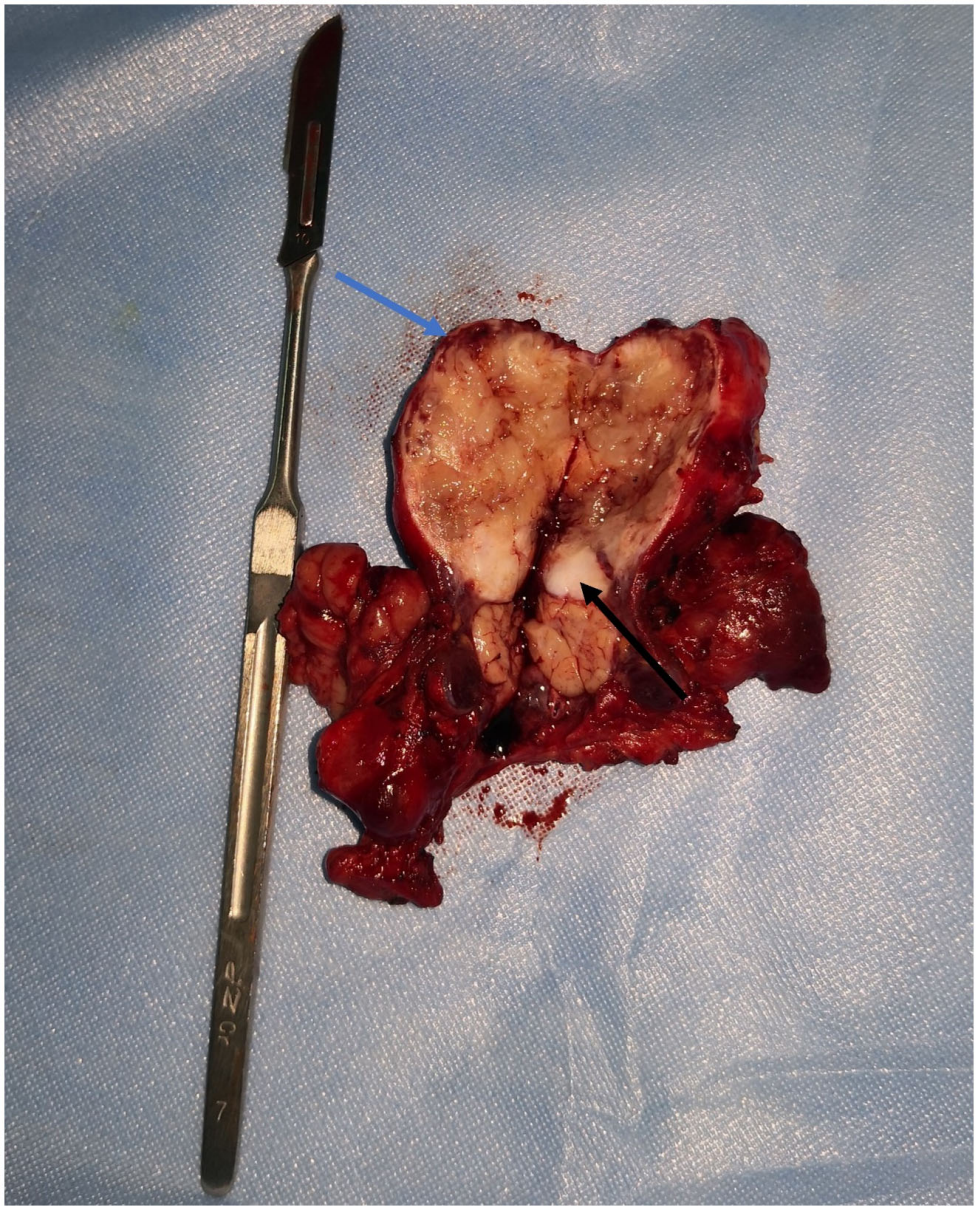
Lesion surgically excised. The submandibular mass had a grey and white appearance with multifocal hemorrhagic areas on the cut surface. It was surrounded by tissues similar to capsule (blue arrow) and the attached salivary gland tissue (black arrow) was observed.

**Figure 3:**
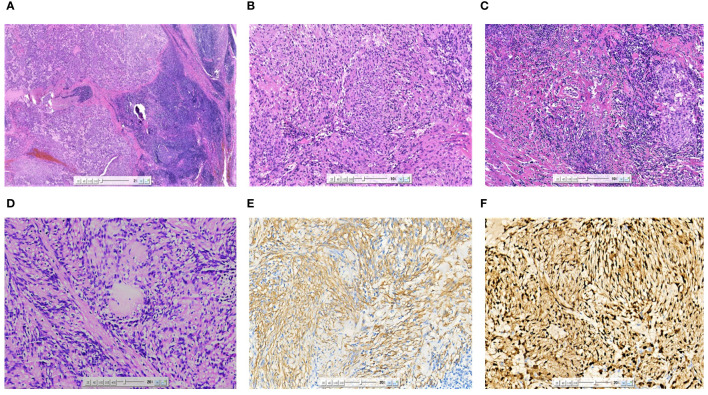
Microscopic image **(A)** of the excised specimen showing the tumor was located in the lymph nodes, and residual lymphoid tissue could be seen at the edge (HE×20). The tumor cells were arranged in a fence-like and woven pattern **(B)** (HE×100). The surrounding area of the tumor was accompanied by significant hyaline degeneration **(C)** (HE×100). The characteristic morphological asbestos-like fibrous nodules **(D)** (HE×200). SMA **(E)** was partially expressed in tumor cells (IHC×200). Cyclin-D1 **(F)** was diffusely expressed in tumor cells (IHC×200).

The patient underwent surgical excision of the lesion. Grossly, the submandibular lesion was well-delimited and firm and had a gray-and-white appearance with multifocal hemorrhagic areas on the cut surface. It was surrounded by tissues similar to the capsule, and the attached salivary gland tissue was observed (**Figure 2**). Microscopic examination revealed that nearly all the biopsy samples comprised fascicles and amianthoid fibers, and the spindle cells were arranged in a fence-like and woven-like manner, without cellular atypia. Significant hyaline degeneration was observed in the surrounding area of the tumor. Residual lymphatic tissue was observed at the edge of the tumor. Immunohistochemical analysis revealed that the tumor cells were partially positive for smooth muscle antibody and diffusely positive for cyclin D1, which was consistent with the manifestations of IPM in the lymph nodes (**Figure 3**). The patient was followed up for 18 months, and no signs of recurrence were observed.

## 3 Discussion

IPM, also known as an intranodal hemorrhagic spindle-cell tumor with amianthoid fibers (5), is a rare benign primary mesenchymal neoplasm that originates from differentiated smooth muscle cells and myofibroblasts. These tumors originate from the lymph nodes, most commonly in the inguinal lymph nodes. IPM is more common in middle-aged people; however, it can also affect various age groups (19–71 years old) (1). Only one case was found in infants (6). IPM is more common in men than in women (2:1), and there is no racial tendency. It has also been reported in white, black, and Asian populations (2). Typical clinical manifestations include unilateral, less painful, single, hard, and movable masses. As a benign lesion, IPM can be cured by surgical resection (3). The recurrence rate is approximately 6% (2), and local recurrence is very rare (3).

Fine-needle aspiration of the mass may help identify specific cellular patterns. IPM has obvious histological, immunohistochemical, and electron microscopic characteristics. It is characterized by the proliferation of spindle cells, hemosiderin-containing tissue cells, and amianthoid fibers in the lymph nodes. IPM has five histological features (2): (a) The remaining lymph node parenchyma is compressed against the capsule, (b) IPM comprises bland-looking spindle cells with abundant areas of nuclear palisading, (c) areas of intraparenchymal hemorrhage and extravasation of erythrocytes between spindle cells are observed, (d) collagenous bundles of so-called amianthoid fibers are distributed throughout the lesion, and (e) extracellular and intracellular fuchsinophilic bodies are present. According to the relevant literature, thick pseudocapsules can be observed around the tumor, mostly accompanied by hyaline degeneration. The tumor was composed of cells with consistent morphology and rare mitotic figures (7). In some cases, calcification can occur in the center of asbestos-like fibers and can result in metaplastic bone formation (8). The immunohistochemical features of IPM help in the diagnosis and exclusion of other soft tissue tumors with nuclear fences, spindle cells, and pigments. Spindle cells were stained with smooth muscle actin, vimentin, cyclin D1, and b-catenin, with a low Ki-67 proliferative index of <5% (9). Cyclin D1 is a characteristic marker of IPM, and nearly all IPM cases are positive for cyclin D1. A study suggested that mutational activation of the β-catenin gene is a key event in the pathogenesis of IPM (7).

Chief radiological investigations include static and functional US (10), and computed tomography can also be used to characterize the lesion to exclude the probability of a different origin or from nearby structures; however, they lack specificity. MRI offers the advantages of high resolution for soft tissues and can clearly visualize the extension of the tumor and adjacent tissues. Few studies have reported the MRI characteristics of IPM. In the presented case, MRI revealed certain characteristics, i.e., a mildly heterogeneous lesion with a hypointense rim demonstrating minimal enhancement following contrast administration.

In this case, the MRI and pathology findings were interpreted as follows: a thick collagenous capsule can be seen around the tumor, often accompanied by glassy degeneration, and a small amount of lymphoid tissue and sinus-like structures can be seen at the edge, which led to the hypointense rim on T2WI. The tumor cells were abundant under the microscope and densely arranged in a fence-like arrangement. The rich tumor cells led to limited diffusion, which was manifested as an increased signal on DWI and a decreased signal on ADC. The interstitial fiber components of the nodules inside the lesion were abundant, the contrast agent cleared slowly, and the TIC gradually increased.

IPM is mainly differentiated from lymphoepithelial carcinoma (LEC) and mucoepidermoid carcinoma (MEC). LEC occurs in women aged 40–50 years. MRI of the submandibular gland LEC mostly shows a lobulated, homogeneous mass with an uneven edge or partially uneven edge, which can be infiltrated along the gland into a cast. It usually shows an equal signal on T1WI, a low signal on T2WI, and moderate enhancement on the enhanced scan (11). Cystic degeneration and calcification in LEC are rare and usually manifest as a relatively uniform signal intensity (12). MEC, a malignant epithelial neoplasm that manifests as a cystic or solid mass, is the most common malignant salivary gland carcinoma (13). The mean age at presentation was 45 years, with slight female predominance. On MRI, MEC tended to have low-to-intermediate signal intensities on T1WI and T2WI. On MRI, low-grade MECs have signal intensities that are indistinguishable from those of pleomorphic adenomas. High-grade lesions have indistinct infiltrating margins and may destroy the salivary ducts (14). In addition, IPM can also be distinguished from schwannoma. Schwannomas contain spindle cells with nuclear palisading (15). The tumor is isointense on T1WI and isointense or hyperintense on T2WI. The specific MRI sign of schwannoma was the target sign, which could be seen as the target sign with high signal around and low signal in the center on T2WI or enhanced scan sequence. Some cases also showed that the enhanced scan of schwannoma showed obvious uneven enhancement (16, 17).

## 4 Conclusion

We reported a rare case of IPM in the submandibular gland region, which is a benign primary mesenchymal tumor originating from differentiated smooth muscle cells and myofibroblasts in the lymph nodes. In IPM, spindle cells, hemosiderin-containing tissue cells, and amyloid fibrils proliferate in the lymph nodes. Although its behavior is benign, to the rarity and the feature’s similarities with other benign and malignant mesenchymal neoplasms, it is often difficult to identify. In this paper, this uncommon origin location may provide an opportunity to reconsider other sites of IPM except inguinal, and the comparison between MRI features and pathological characteristics provides a reference for its differential diagnosis.

## Data availability statement

The original contributions presented in the study are included in the article/supplementary material. Further inquiries can be directed to the corresponding authors.

## Ethics statement

The studies involving humans were approved by the ethics committee of Jining No.1 People’s Hospital (KYLL-202309-168). The studies were conducted in accordance with the local legislation and institutional requirements. Written informed consent was obtained from the participant/patient(s) for the publication of this case report.

## Author contributions

HZ: Conceptualization, Writing – original draft. MW: Investigation, Writing – original draft. LL: Writing – original draft, Investigation, Data curation. SS: Project administration, Writing – review & editing, Supervision, Funding acquisition. NZ: Writing – review & editing, Resources, Project administration, Data curation.
